# Accelerating innovation and ensuring the thoughtful withdrawal of lifeline medicines for people living with diabetes in Asia

**DOI:** 10.1111/jdi.70063

**Published:** 2025-05-20

**Authors:** Yutaka Seino, Daisuke Yabe, Sung Hee Choi, Chih‐Cheng Hsu, Chien‐Ning Huang, Altaisaikhan Khasag, Kathryn Tan, Kohjiro Ueki, Yuichiro Yamada, Zhanay A Akanov, Takashi Kadowaki

**Affiliations:** ^1^ Kansai Electric Power Hospital Osaka Japan; ^2^ Kansai Electric Power Medical Research Institute Kyoto Japan; ^3^ Department of Diabetes, Endocrinology and Nutrition Kyoto University Graduate School of Medicine Kyoto Japan; ^4^ Seoul National University College of Medicine Seoul Korea; ^5^ Department of Internal Medicine Seoul National University Bundang Hospital Seongnam Korea; ^6^ National Center for Geriatrics and Welfare Research National Health Research Institutes Zhunan Taiwan; ^7^ School of Medicine Chung Shan Medical University Taichung Taiwan; ^8^ Department of Internal Medicine, School of Medicine Mongolian National University of Medical Sciences Ulaanbaatar Mongolia; ^9^ Department of Medicine, School of Clinical Medicine The University of Hong Kong Hong Kong Hong Kong; ^10^ Diabetes Research Center National Institute of Global Health and Medicine, Japan Institute for Health Security Tokyo Japan; ^11^ Kazakh Scientific Research Institute of Cardiology and Internal Diseases Almaty Kazakhstan; ^12^ Toranomon Hospital Tokyo Japan

## Abstract

Coordinated regional action is urgently needed to safeguard insulin access in Asia amid global product realignment. This editorial emphasizes the ethical imperative and policy frameworks required to balance innovation with equitable, uninterrupted diabetes care.

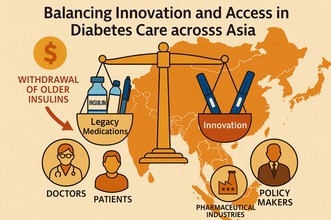

At the 17th Scientific Meeting of the Asian Association for the Study of Diabetes (AASD), held in conjunction with the 46th Annual Meeting of the Diabetes Association and Endocrine Society of the Republic of China, insulin supply instability emerged as a pressing issue with continent‐wide implications. The round table discussion, chaired by Professor Yutaka Seino (Kansai Electric Power Hospital/Kansai Electric Power Medical Research Institute) who is Chair of AASD, brought together expert voices from across Asia to discuss the multifaceted crisis in insulin access and formulate a regional response rooted in collaboration and equity.

## THE GLOBAL CONTEXT AND ASIAN REALITIES

Mr. Kasper Bødker Mejlvang of Novo Nordisk Pharma Ltd., Japan, opened the session by addressing the company's decision to phase out older‐generation insulin delivery devices starting in 2026. Despite Novo Nordisk supplying insulin to over 45 million people worldwide and investing over USD 7.5 billion to expand production capacity, he admitted the system remains under strain. Nearly 2,000 product variants—many customized for individual markets—have made scaling difficult and costly.

This global realignment is intended to streamline operations, but in Asia, the consequences could be profound. The region hosts over 40% of the world's diabetes population, and each country/region face distinct clinical, regulatory, and economic challenges. What emerges is not a single crisis, but many interwoven challenges that require nuanced, context‐aware responses.

The rising prevalence of diabetes, particularly among aging populations and low‐ to middle‐income groups, has only deepened the urgency. In many Asian countries, public healthcare systems are the backbone of chronic disease management, making sudden changes in drug availability particularly disruptive. Without region‐specific planning, these shifts could lead to therapy gaps, increased healthcare costs, and compromised patient outcomes.

## PERSPECTIVES FROM JAPAN AND KOREA: UNIQUE NEEDS, SHARED VULNERABILITIES

In Japan, Professor Kohjiro Ueki (Japan Institute for Health Security) voiced concern over the discontinuation of essential insulins like insulin detemir, especially for use in gestational diabetes. He stressed that the absence of domestic biosimilar manufacturers places the country in a precarious position. Similarly, Professor Seino referenced examples from Kazakhstan, shared by Professor Zhanay A. Akanov (Kazakh Scientific Research Institute of Cardiology and Internal Diseases), to highlight that sudden product withdrawals could seriously disrupt clinical care in Japan. He emphasized the ethical tension between maintaining affordability and withdrawing affordable products. Professor Seino called on Novo Nordisk to conduct country‐specific dialogues and to view access as a shared responsibility. Korea's experience, shared by Professor Sung‐Hee Choi (Seoul National University Bundang Hospital), echoed many of these concerns. The abrupt withdrawal of vial‐type insulin in 2011 had already strained the Korean healthcare system. Now, facing the discontinuation of mixed insulin products by 2026, Professor Choi urged Novo Nordisk to adopt a slower, more consultative approach. She emphasized that older and socioeconomically vulnerable patients often rely on these legacy insulins for continued care. She proposed structured hearings and greater transparency to ensure smooth transitions. Professor Choi also acknowledged Novo Nordisk's legacy of principled practice, noting, “For over a century, your company has followed a steady, ethical path in advancing diabetes care. That history is why we are asking for patience and planning today.”

## WARNINGS FROM HONG KONG AND TAIWAN: INFRASTRUCTURE AND ECONOMIC BARRIERS

Professor Kathryn Tan (The University of Hong Kong) warned that while newer analogues are medically beneficial, their higher cost and distribution constraints would significantly disrupt Hong Kong's public health infrastructure. Clinics are understaffed, budgets are fixed, and the absence of domestic biosimilar options makes the switch logistically impossible without transitional funding. She explained that the budget for the upcoming fiscal year had already been set and does not accommodate the increased cost of new insulins. Transitioning patients would also require additional medical appointments and staff time—resources that are currently in short supply. Professor Tan stressed that governments and pharmaceutical companies must share the financial burden of transitions. Professor Chih‐Cheng Hsu (National Health Institute of Republic of China) highlighted the regulatory and pricing issues that make insulin supply unsustainable. He proposed designating insulin as an essential medicine to ensure uninterrupted access and called for governments to create longer procurement contracts to allow for strategic planning and stability. He also warned that the industry's increasing focus on glucagon‐like peptide‐1 (GLP‐1) therapies could risk deprioritizing insulin at a time when demand remains critical. Professor Hsu urged governments to consider price protections for essential medicines and to reduce reliance on short‐term contracts that leave suppliers with little long‐term incentive. He also suggested broader public–private partnerships, noting that collaboration—not confrontation—is the only way forward.

## MONGOLIA'S STRUGGLES: THE FRAGILITY OF SOLE‐SUPPLIER DEPENDENCE

Professor Altaisaikhan Khasag (Mongolian National University of Medical Sciences) painted a vivid picture of Mongolia's dependence on a single insulin supplier—Novo Nordisk. In a country where political turnover is frequent and communication systems underdeveloped, advance warnings about supply changes often fail to reach healthcare providers in time. Professor Khasag called for Mongolia's integration into the East Asian supply and training framework, urging leadership from Korea or Japan to ensure information‐sharing. He also noted that Mongolia's current supply route—through Kazakhstan and other former Soviet states—is outdated and inefficient. The younger generation of Mongolian clinicians no longer speaks Russian, the language used in most training and documentation. Realignment toward the East Asian framework could better reflect Mongolia's current needs. Mr. Mejlvang acknowledged these gaps and committed to improving communication pipelines, not only through ministries but also directly with medical associations. He reaffirmed Novo Nordisk's policy of not abandoning any market, no matter how small or geopolitically unstable. He cited the company's continued presence in Ukraine and other high‐risk areas as evidence of this commitment.

## ADVOCACY FROM REGIONAL BODIES: AASD AND IDF‐WPR

Speaking for the AASD, Professor Daisuke Yabe (Kyoto University) offered a critique of one‐size‐fits‐all strategies. He argued that Asia's diabetes phenotype—marked more by impaired insulin secretion than insulin resistance—requires insulin as an important therapy. Professor Yabe criticized the withdrawal of the FlexTouch delivery device for insulin aspart—a formulation widely used in Japan—attributing the decision to the soaring global demand for the GLP‐1 receptor agonist semaglutide. He urged Novo Nordisk to consider offering GLP‐1 receptor agonists in penfill or vial formats to alleviate pressure on device supply and facilitate smoother transitions. Professor Takashi Kadowaki (Toranomon Hospital), Chair of the International Diabetes Federation Western Pacific Region (IDF‐WPR), echoed these concerns, stating that the insulin supply issue had been unanimously recognized as a top priority at the most recent Executive Board meeting. He urged Novo Nordisk to shift from global uniformity to regional precision, arguing, “Strategies must reflect the realities of each country. What works in the U.S. may not be feasible in Asia.” Professor Kadowaki added, “This is a call to action—not just for industry, but for policymakers, researchers, and associations. We must build regionally responsive frameworks rooted in patient needs, not just market logic.”

## ETHICAL REFLECTIONS AND CORPORATE RESPONSIBILITY

Throughout the session, questions of ethics, responsibility, and corporate values were woven into the conversation. Professor Chien‐Ning Huang (Chung Shan Medical University), President of the 17^th^ AASD scientific meeting, reminded participants that while the patient base for older insulins may be small, their lives carry equal value. “Even one overlooked patient is one too many,” he emphasized. Professor Choi appealed to Novo Nordisk's century‐long legacy of ethical leadership, urging that all product withdrawals be preceded by hearings, surveys, and structured dialogues in each country. “It's not about protest—it's about partnership,” she said. Novo Nordisk's representatives responded with empathy and a pledge to improve. Mr. Mejlvang stated that rapid product consolidation, while operationally necessary, must never outweigh the need for compassion and collaboration. Dr. Marcin Zychma of Novo Nordisk shared that employees within Novo Nordisk also feel the weight of these decisions deeply. “We do not take these steps lightly,” he affirmed. Dr. Zychma recalled the evolution of diabetes care, from the early Diabetes Control and Complications Trial to modern GLP‐1 therapies, and emphasized that while progress is essential, the transition must be managed carefully. “Patients are not just numbers—they're lives. And transitions must never cost them their stability,” he said.

## A REGIONAL ROADMAP FOR THE FUTURE

In response to collective calls for action, Mr. Mejlvang outlined a path forward. Novo Nordisk is open to providing active pharmaceutical ingredients for older‐generation insulins, enabling regional or local biosimilar producers to step in. The company also supports public–private partnerships, long‐term procurement planning, and customized strategies for each market. He emphasized the recent inclusion of an Asia‐focused senior executive in the company's global leadership team—an effort to better incorporate regional considerations into worldwide strategies. He promised improved transparency, better communication channels, and more participatory planning. Novo Nordisk also expressed openness to revisiting the idea of a penfill device strategy in Asia, recognizing that this format could ease the burden on both people living with diabetes and healthcare systems. The company reiterated its long‐standing commitment to affordability, even if that means maintaining certain low‐profit formulations in key markets.

## CONCLUSION: COLLECTIVE RESPONSIBILITY

Professor Seino concluded the roundtable by reiterating that the region's insulin crisis is not merely a supply‐chain problem, but a test of moral priorities. Asia's healthcare systems are diverse, yet interconnected. A disruption in one country often has ripple effects in another. The roundtable discussion closed with a clear consensus: regional collaboration, early and consistent communication, and context‐aware policy frameworks are critical to safeguarding insulin access across Asia. From Tokyo to Taipei, Ulaanbaatar to Seoul, and Almaty to Hong Kong, the message was consistent—solutions must be as diverse and thoughtful as the communities they intend to serve. Insulin access is not just about medicine; it is about justice, responsibility, and the will to listen before acting. The future of diabetes care in Asia depends on our collective ability to meet that standard.

## FUNDING

The authors received no financial support relevant to this article.

## DISCLOSURE

YS has received research funding/grants from Nippon Boehringer Ingelheim Co., Ltd., ARKRAY Marketing, Inc., Taisho Pharmaceutical Co., Ltd., Novo Nordisk Pharma Ltd., Terumo Corporation, and Sumitomo Pharma Co.; and consulting/lecture fees from Taisho Pharmaceutical Co., Ltd., Nippon Becton Dickinson Company, Ltd., Novo Nordisk Pharma Ltd., Eli Lilly Japan K.K., Sumitomo Pharma Co., Ltd., and Ono Pharmaceutical Co., Ltd. DY has received consulting/lecture fees from Eli Lilly Japan K.K., Kyowa Kirin Co., Ltd., Nippon Boehringer Ingelheim Co., Ltd., Novo Nordisk Pharma Ltd, Sanofi K.K., and Sumitomo Pharma Co., Ltd.; and research funding/grants from Arkray Inc., the Japan Association for Diabetes Education and Care, Nippon Boehringer Ingelheim Co., Ltd., Novo Nordisk Pharma Ltd., Taisho Pharmaceutical Co., Ltd., and Terumo Corporation. SHC has received research funding/grants from Eli Lilly and Company, Novo Nordisk Pharma Ltd., and AstraZeneca, and consulting/lecture fees from Jeil Pharmaceutical Co, Ltd, Daewoong Pharmaceuticals Co., Ltd, and Viatris. CCH has received research funding/grants from AstraZeneca, Eli Lilly, Sanofi, Amgen, and Novo Nordisk Pharma Ltd; and lecture fees from AstraZeneca and Sanofi. CNH has received research funding/grants from AstraZeneca, Eli Lilly and Company, Novo Nordisk Pharma Ltd., Sanofi; and consulting/lecture fees from Abbott Laboratories, AstraZeneca, Bayer, Boehringer Ingelheim Co., Ltd., Eli Lilly and Company, Medtronic, Novartis, Novo Nordisk Pharma Ltd., Sanofi. AK has received consulting/lecture fees from Novo Nordisk. KT has received consulting/lecture fees from Amgen, AstraZeneca, Bayer, Boehringer Ingelheim, Daiichi Sankyo, Eli Lilly, Novo Nordisk, and Sanofi. KU has received grants and endowments from Astellas, Eli Lilly, Kyowa Kirin, Mitsubishi‐Tanabe, MSD, Nippon Boehringer Ingelheim, Novartis, Novo Nordisk, Ono, and Sumitomo; lecture fees from AstraZeneca, Kyowa Kirin, Mitsubishi Tanabe, MSD, Nippon Boehringer Ingelheim, Novo Nordisk, Ono, Sanofi, Sumitomo, and Taisho. YY has consulting/lecture fees from Novo Nordisk Pharma Ltd., Sumitomo Pharma Co., Ltd., Teijin Pharma Ltd., and Ono Pharmaceutical Co., Ltd. ZAA has received consulting/lecture fees from Abbott Laboratories, AstraZeneca, Boehringer Ingelheim Co., Ltd., Eli Lilly and Company, Medtronic, Novartis, Novo Nordisk Pharma Ltd., and Sanofi. TK has received research funding/grants from Daiichi Sankyo Co., Ltd., Sumitomo Pharma Co., Ltd., Nippon Boehringer Ingelheim Co., Ltd.; and consulting/lecture fees from Taisho Pharmaceutical Co., Ltd., Sumitomo Pharma Co., Ltd., Takeda Pharmaceutical Co., Ltd., Mitsubishi Tanabe Pharmaceutical Co., Ltd., Eli Lilly Japan K.K., Nippon Boehringer Ingelheim Co., Ltd., Novo Nordisk Pharma Ltd., Abbott Japan LLC., Teijin Pharma Ltd. YS, DY, KT, UK, and TK are Editorial Board members of the Journal of Diabetes Investigation and co‐authors of this article. To minimize bias, they were excluded from all editorial decision‐making related to the acceptance of this article for publication.

Approval of the research protocol: N/A.

Informed consent: N/A.

Registry and the registration no. of the study/trial: N/A.

Animal studies: N/A.

## AUTHOR CONTRIBUTIONS

YS and DY contributed to the writing of the manuscript and approved the version to be published. SHC, CCH, CNH, AK, KT, KU, YY, ZAA, and TK critically reviewed and revised the manuscript for intellectual content. YS and DY are the guarantors of this work.

## Data Availability

Data sharing not applicable to this article as no datasets were generated or analysed during the current study.

